# Accuracy of Fetal Weight Estimation by Ultrasonographic Evaluation in a Northeastern Region of India

**DOI:** 10.1155/2021/9090338

**Published:** 2021-12-20

**Authors:** Ranjumoni Konwar, Bharati Basumatary, Malamoni Dutta, Putul Mahanta

**Affiliations:** ^1^Radiology, Fakhruddin Ali Ahmed Medical College and Hospital, Barpeta 781301, Assam, India; ^2^Anatomy, Assam Medical College and Hospital, Dibrugarh 786002, Assam, India; ^3^Forensic Medicine and Toxicology, Assam Medical College and Hospital, Dibrugarh 786002, Assam, India

## Abstract

**Methods:**

The cross-sectional study included 100 pregnant women aged 20–45 years from the Kamrup district admitted to Guwahati Medical College and Hospital, Guwahati, Assam. The data were analyzed using Microsoft Excel and SPSS version 16. The EFW at term was calculated using Shepard's formula and Hadlock's formula. Differences in means are compared using the one-way ANOVA or Kruskal–Wallis test and paired *t*-test. The accuracy of the two procedures was evaluated using mean absolute error (MAE) and mean absolute percentage error (MAPE). A *p* value<0.05 was considered significant.

**Results:**

The present study included 100 pregnant women aged 21–38 years with term or postterm pregnancies subjected to ultrasonographic evaluation within 72 hours of delivery. The mean (±s.d.) EFW by Shepard's formula was 2716.05 (±332.38) g and Hadlock's formula was 2740.44 (±353.23) g, respectively. For Hadlock's formula, MAE ± s.d. was found to be higher (overall 84.59 ± 76.54) specifically in the weight category less than 2500 (106.42 ± 88.11) as compared to Shepard's (overall MAE ± s.d = 79.86 ± 64.78, and among ABW < 2500 g, MAE ± s.d = 65.04 ± 61.02). The overall MAPE of Hadlock's formula was 3.14% and that for Shepard's formula was 2.91%, and the difference was not statistically significant. Both Shepard's formula and Hadlock's formula had a sensitivity of 92.85% in detecting IUGR, but Hadlock's method had higher specificity (66%), higher PPV (86.67%), and higher NPV (80%).

**Conclusion:**

The ultrasonographic evaluation of fetal weight helps predict fetal birth weight precisely and can influence obstetric management decisions concerning timing and route of delivery, thus reducing perinatal morbidity and mortality.

## 1. Introduction

Estimation of fetal weight in utero had formed a significant component in obstetrical practices, ever since the inception of ultrasonography pioneered by Dr. Ian Donald in 1958 [1]. Fetal weight estimation plays a vital role in many obstetrical decisions regarding intrauterine growth monitoring, delivery, as well as determining high-risk pregnancies [2, 3].

Fetal weight is considered an independent risk factor of high perinatal morbidity and mortality. Both low and high fetal weight at delivery may lead to complications in labor and delivery to both the child and mother [2–4].

The perinatal complications associated with low birth weight are fetal prematurity and IUGR, a growth disorder of the fetus defined as a fetal weight below the 10^th^ percentile for age, which is a significant risk factor of neonatal morbidity and mortality [5].

Macrosomia is defined as a fetal weight above 4000 g or a birth weight above the 90^th^ percentile for gestational age. Inaccuracy in the estimation of fetal weight may also increase the risk in management and during delivery, specifically in suspected macrosomia [6–9].

Before the availability of ultrasound, conventional methods such as palpation of the maternal circumference and measurement of the maternal abdominal circumference and uterine height were used to estimate fetal weights. Though the traditional methods are easy to use, several maternal factors may affect assessment abilities, such as myomas, obesity, multiple gestations, and the amount of amniotic fluid. Hence, the assessments by the conventional methods may be inaccurate sometimes [2, 10].

Modern sonographic predictions are based on regression algorithms using various combinations of fetal parameters such as head circumference (HC), the length going around the baby's head, abdominal circumference (AC), the length going around the baby's belly, biparietal diameter (BPD), the diameter of the baby's head, and femur length (FL), the length of the femur in the baby's leg. Earlier, many authors suggested various formulas for fetal weight estimation using single fetal parameters [11–13]. Subsequently, utilization of two parameters for fetal weight measurement, including BPD and AC, was introduced by few authors since 1970s [14–16]. Hadlock et al. [17] proposed an improved formula for fetal weight estimation using HC, AC, FL, and BPD. Various combinations of ultrasonically derived measurements were tried and tested by many authors with complex mathematical expressions for estimating fetal weights [18].

The present study aims to determine the usefulness and accuracy of estimating fetal weight at term pregnancies using sonographic measurements by two available formulas: Shepard's formula and Hadlock's formula, with particular reference to suspected IUGR macrosomia in a northeastern city of India.

## 2. Materials and Methods

A cross-sectional study includes 100 pregnant women with term and postterm pregnancies from the Kamrup district of Assam admitted to Guwahati Medical College and Hospital aged 20–45 years. Participants with a gestational age of 37–40 weeks and who were expected to deliver less than 72 hours of sonographic examination were included. Viable fetuses of all weight categories were included concerning suspected IUGR babies. Suspected fetuses of having brachycephaly (cephalic index > 85) and dolichocephaly (CI < 75) were excluded.

The sample size was determined using the Windows version (WINPEPI version 11.65) of the PEPI suite of programs for epidemiologists. The sample size of 100 is sufficient to estimate a difference in mean weight of 100 g with a 5% level of significance and 80% power considering standard deviation of EFW by Hadlock's formula as 420 g and by Shepard's formula as 0.538 g and correlation coefficient of 0.77 [19].

The sonographic evaluation was performed using Siemens Sonoline 2, a real-time grayscale scanner using a 3.5 MHz transducer. A linear array probe was preferably used for the examination. Fetal weight estimation was derived from Shepard's formula = BPD/AC and Hadlock's formula = AC/FL. BPD is measured at the widest portion of the skull from the outer edge of the proximal skull table to the inner edge of the distal skull table perpendicular to the middle echo complex using electronic callipers. The AC was measured in the transverse plane perpendicular to the long axis of the fetal aorta or spine at the level of the portoumbilical vein complex within the liver. FL is measured as the linear distance between two calcified diaphyseal ends of the femur, which corresponds anatomically from the greater trochanter of the femur to the distal metaphysis. All the sonographic measurements were recorded in millimetres (mm). Estimation of fetal weights was done by referring to established charts derived from two published formulas, i.e., Shepard's formula that used the combination of BPD and AC is represented as

(1) $$\begin{array}{c} \text{EFW}:\;\log_{10}\left(\text{BW}\right)=-1.7492+0.166\left(\text{BPD}\right)+0.046\left(\text{AC}\right)-2.646\left(\text{AC}+\text{BPD}\right)/1000. \end{array}$$

Hadlock's formula that used the combination of AC and FL is represented as

(2) $$\begin{array}{c} \text{EFW:}\log_{10}\left(\text{BW}\right)=1.3598+0.051\left(\text{AC}\right)+0.1844\left(\text{FL}\right)-0.0037\left(\text{AC}\times \text{FL}\right). \end{array}$$

Each study participant was thoroughly assessed before ultrasound evaluation, including patient history, clinical examination, and routine laboratory examination.

### 2.1. Statistical Analysis

The paired *t*-test and one-way ANOVA or Kruskal–Wallis test were performed to test the difference in means. Correlation analysis was done using scatter plots. The accuracy of the sonographic estimation methods was determined by computing the mean absolute error as MAE = mean|EFW − ABW| and mean absolute percentage error as MAPE = mean|(EFW − ABW/ABW) × 100)|. The accuracy of the formulas in detecting IUGR was assessed using sensitivity analysis. The data were analyzed using Microsoft Excel and SPSS version 16. A *p* value <0.05 was considered significant. Prior collection of the ethical data approval was taken from the Ethics Committee of Guwahati Medical College, Guwahati, Assam, and the participant's informed consent.

## 3. Results

The study participants' age range was 21–38 years. Most participants (59%) were from urban areas and belonged to the middle or lower class economic background (75%). Also, 20% had suspected IUGR.

Among the 100 pregnancies, 30% each were primigravida and primipara and 40% were multiparous women. The majority, 62%, deliveries were spontaneous, and 31% were by the lower segment cesarian section (LSCS). The mean and standard deviation (s.d.) ABW of the newborns was 2740.35 ± 354.19 gm. Among the newborns, 48% were male and 52% were females. Eighty-four babies had weight appropriate for gestational age (AGA), 14 were small for gestational age (SGA), and only 2 were large for gestational age (LGA). Mean maternal weight was found significantly different (*p* value <0.01) among the AGA, SGA, and LGA groups (Table 1).

The mean (±s.d.) EFW by Hadlock's formula was found to be 2740.44 (353.23), which was close to the mean (±s.d.) ABW of 2740.35 (354.19), and the paired *t*-test revealed no significant difference between mean ABW and mean EFW by Hadlock's formula. But in our present study, the mean EFW by Shepard's formula was found to be significantly different (*p* value 0.017) from mean ABW, as given in Table 2.

**Figure 1:**
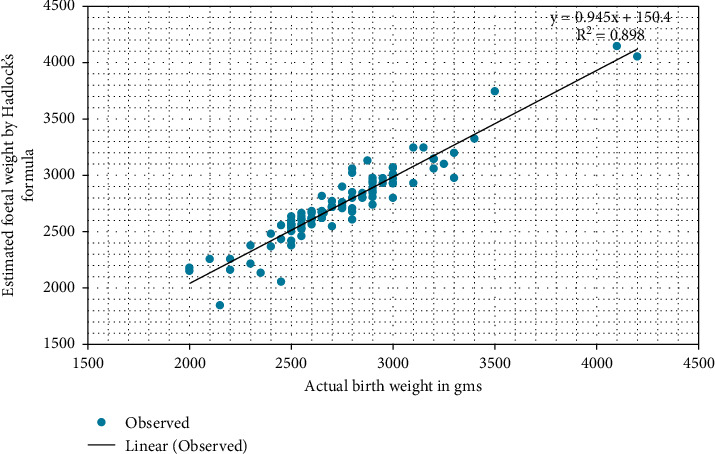
Correlation between EFW (Hadlock's) and ABW.

**Figure 2:**
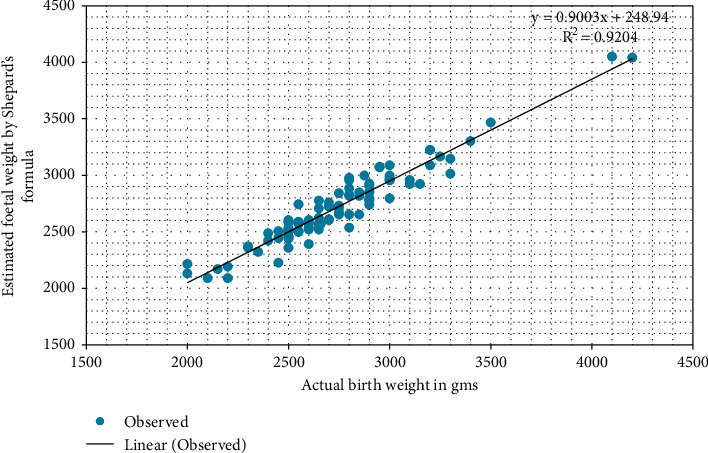
Correlation between EFW (Shepard's) and ABW.

The EFWs by Hadlock's formula plotted against ABW of the newborns reveal a significant positive correlation (*r* = 0.948, *p* value <0.01), as shown in Figure 1. Similarly, as shown in Figure 2, the EFWs by Shepard's formula plotted against ABW show a linear relationship with a significant positive correlation (*r* = 0.96, *p* value <0.01).

Both formulas estimated an equal number of cases in the weight category 2501–3000 g (67%). In the 2001–2500 g weight category, Shepard's formula could assess more cases than Hadlock's formula. Also, Shepard's formula predicted the exact number of patients in more than 3500 g weight groups. Both the procedures predicted 2 macrosomic birth, as given in Table 3.

The MAE for Hadlock's formula was higher than that for Shepard's formula. MAE for Hadlock's procedure was highest in the below 2500 g ABW weight group with MAPE of almost 4.6%. The overall MAPE of Hadlock's formula was 3.14% and that for Shepard's formula was 2.91%. The difference was not statistically significant, as given in Table 4.

### 3.1. Sonological Detection of IUGR Cases

Both the formulas correctly predicted 13 IUGR cases out of a total of 15 proved IUGR cases and had 92.8% sensitivity in detecting IUGR; however, Shepard's formula had a lower specificity as compared to Hadlock's formula, as given in Table 5.

### 3.2. Sonological Detection of Macrosomia Cases

In our study, two patients were estimated by both formulas. Both the cases were proved to be macrosomia by ABW.

## 4. Discussion

Fetal weight estimation at term may reduce the risk of perinatal mortality and morbidity by predicting IUGR and macrosomia before birth. As fetal weight cannot be measured directly, fetal and maternal anatomical characteristics (BMI, sex, and gestational age) are being used over the years [3, 20].

The age range of the patients was 21–38 years. The present study reveals that out of 14 SGA babies, mothers of 6 had parity 0. Many studies have suggested nulliparity as a factor affecting low birth weight [21–23]. Also, mean maternal weight was significantly different among AGA, SGA, and LGA groups (*p* value<0.01) with a proportional increase in ABW with maternal weight implying maternal weight gain as a significant factor affecting the weight gain of the baby inside the womb [24–26].

The mean ABW in the present study was 2740.35 (354.19) g which is concordant with many other studies conducted in different parts of India [27–29]. Mean EFW by Shepard's formula was significantly different from mean ABW, indicating a fair underestimation of ABW by Shepard's formula [30, 31]. EFW estimated from both the procedures yielded an incredibly positive correlation with ABW of more than 0.9 coincident with other studies [3, 32, 33]. For Hadlock's formula, MAE was higher, specifically in the weight category less than 2500 g compared to Shepard's. MAE in our study is relatively lower than several other studies [3, 34, 35]; however, the results suggest that the accuracies of EFW formulas changed with different weight categories [34]. The overall MAPE of Hadlock's formula was 3.14% and that for Shepard's formula was 2.91%, and the difference was not statistically significant. The MAPE in our study was lower than other studies [36, 37], suggesting the formula's usefulness in estimating the fetal weights at term in our local population.

Both the formulas had a sensitivity of 92.85% in detecting IUGR, but Hadlock's method had higher specificity (66%). Various studies recommended different Hadlock's formulas as efficient in predicting IUGR with high sensitivity and specificity [38, 39]. While in contrast to our findings, Shepard's formula was reported to have very low sensitivity and specificity in detecting IUGR in a review [40].

In the current study, 20 cases were suspected IUGR, out of which 15 were proved IUGR cases. Both the formulas correctly predicted 13 IUGR cases. There were 8 mothers diagnosed with pregnancy-induced hypertension, 2 of which have been associated with IUGR babies. Studies have identified hypertensive disorder in mothers as one of the most typical causes of IUGR due to placental insufficiency [41–43]. In the present study, we have also used the “lcm rule” to assess amniotic fluid status [44]. The criterion for oligohydramnios is the largest vertical fluid pocket to be less than 1 cm. In the present study, 6 pregnant mothers were found to have less amniotic fluid, out of which 5 (38.46% of proved IUGR) mothers have given birth to IUGR babies. Thus, the predictive accuracy of sonography in the assessment of oligohydramnios in IUGR of our study was 83%, which is comparable to the observations of Manning et al. [44].

In the present study, there were two cases of proven macrosomia, out of which one was a known diabetic case while the other one was presenting as a breach with the inadequate pelvis. Both cases had undergone LSCS after sonographically they were diagnosed to have macrosomic fetuses. Also, in the present study, out of 6 cases of breech presentation, 5 cases had a fetal weight of greater than 3 kg, who all had undergone LSCS. Thus, the sonographic prediction of fetal weight helped in timely intervention by LSCS in all these cases.

Although EFW by sonography is an infallible tool for modern obstetric practice, its accuracy may comprise intra and interobserver variability. Multiple measurements, picture quality improvement, equipment calibration, proper design, and refinement of measuring methods may help in reducing variability. More research is needed to improve the universal validity and accuracy of fetal weight estimate equations [45].

### 4.1. Limitation

The study considered only two formulas to estimate the EFWs by ultrasonographic measurements.

## 5. Conclusion

Both Shepard's and Hadlock's provide fair estimates of fetal weight with minimal error and may be considered valuable tools in predicting the weight of fetuses in pregnancies in this region.

## Figures and Tables

**Table 1:** Sociodemographic profile of the participants.

Characteristics	Mean (s.d.)	Median	Range	Mean (s.d.) SGA (*n* = 14)	Mean (s.d.) AGA (*n* = 84)	Mean (s.d.) LGA (*n* = 2)	*P* value, K-W test^#^
Maternal age	27.13 (3.34)	26.50	21–38	26.43 (2.34)	27.30 (3.49)	25.00 (1.41)	0.44
Parity^##^		1.00	0–5	0–3	0–5	2	—
Maternal weight	52.89 (3.49)	52.00	48–64	49.57 (1.99)	53.25 (3.21)	61.00 (2.83)	<0.01^*∗*^
Gestational age at delivery	39.64 (1.42)	40.00	37–49	39.71 (0.91)	39.63 (1.50)	39.50 (0.71)	0.93

^*∗*^ *P* value < 0.05 significant. ^#^K-W test, Kruskal–Wallis test for more than two means. ^##^Parity is represented as median and range. s.d., standard deviation; SGA, small for gestational age (SGA); AGA, appropriate for gestational age; LGA, large for gestational age.

**Table 2:** Comparison of mean ABW and mean EFWs by ultrasonography methods.

	*N*	Mean (s.d.)	Minimum	Maximum	Paired *t*	*P* value
ABW	100	2740.35 (354.19)	2000	4200		
EFW by Hadlock	100	2740.44 (353.23)	1847	4145	−0.008	0.994
EFW by Shepard	100	2716.05 (332.38)	2089	4050	2.42	0.017

s.d., standard deviation; ABW, actual birth weight; EFW, estimated fetal weight.

**Table 3:** Distribution EFW and ABW among different weight categories.

Weight category	EFW by Hadlock's formula	EFW by Shepard's formula	ABW
	Frequency (%)	Frequency (%)	Frequency (%)
1500–2000	1 (1.0)	0 (—)	2 (2.0)
2001–2500	15 (15.0)	20 (20.0)	24 (24.0)
2501–3000	67 (67.0)	67 (67.0)	61 (61.0)
3001–3500	14 (14.0)	11 (11.0)	11 (11.0)
3501–4000	1 (1.0)	0 (—)	0 (—)
4001–4500	2 (2.0)	2 (2.0)	2 (2)

ABW, actual birth weight; EFW, estimated fetal weight.

**Table 4:** Accuracy of the different formulas for estimating EFW by the ultrasonic method.

ABW categories		MAE, g (Hadlock's)	MAE, g (Shepard's)	MAPE, % (Hadlock's)	MAPE, % (Shepard's)
≤2500 g	*N* = 26	106.42 ± 88.11	65.04 ± 61.02	4.59 ± 3.89	2.80 ± 2.77
2501–3500 g	*N* = 72	76.39 ± 71.52	84.53 ± 65.85	2.64 ± 2.38	2.96 ± 2.25
>3500 g	*N* = 2	96.00 ± 72.12	104.50 ± 77.07	2.30 ± 1.70	2.50 ± 1.81
Total	*N* = 100	84.59 ± 76.54	79.86 ± 64.78	3.14 ± 2.94	2.91 ± 2.36
		*P* value for the paired *t*-test = 0.54		*P* value for the paired *t*-test = 0.45	

ABW, actual birth weight; *N*, total sample size; MAE, mean absolute error; MAPE, mean absolute percentage error.

**Table 5:** Sonographic evaluation of IUGR.

Diagnostics	No. of cases (%) by Shepard's	No. of patients (%) by Hadlock's
Predicted IUGR	16	15
Proved IUGR	13	13
Missed IUGR	1	1
Wrongly diagnosed IUGR	3	2
Sensitivity	92.86%	92.86%
Specificity	50.0%	66.67%
Positive predictive value (PPV)	81.25%	86.67%
Negative predictive value (NPV)	75.00%	80.00%
Accuracy	80.00%	85.00%

IUGR, intrauterine growth retardation.

## Data Availability

The data used to support the findings of this study are included within the article.
